# Correlating protein function and stability through the analysis of single amino acid substitutions

**DOI:** 10.1186/1471-2105-10-S8-S8

**Published:** 2009-08-27

**Authors:** Yana Bromberg, Burkhard Rost

**Affiliations:** 1 Department of Biochemistry and Molecular Biophysics, Columbia University, 630 West 168^th ^Street, New York, NY 10032, USA; 2 Columbia University Center for Computational Biology and Bioinformatics (C2B2), 1130 St. Nicholas Ave. Rm. 802, New York, NY 10032, USA; 3 NorthEast Structural Genomics Consortium (NESG) and New York Consortium on Membrane Protein Structure (NYCOMPS), Columbia University, 1130 St. Nicholas Ave. Rm. 802, New York, NY 10032, USA; 4 TU Munich, Bioinformatik & Institute for Advanced Studies, Boltzmannstrasse 3, 85748 Garching, Germany

## Abstract

**Background:**

Mutations resulting in the disruption of protein function are the underlying causes of many genetic diseases. Some mutations affect the number of expressed proteins while others alter the activity on a per-molecule basis. Single amino acid substitutions as caused by non-synonymous Single Nucleotide Polymorphisms (nsSNPs) often disrupt function by altering protein structure and/or stability, but can also wreak havoc by directly impacting functional binding sites. Given the experimental three-dimensional (3D) structure of a protein, we can try to differentiate between the "effect on structure/stability" and the "effect on binding". However, experimental 3D structures are available for only 1% of all known proteins; the magnitude of stability change caused by a given mutation is more widely available.

**Results:**

Here, we analyze to which extent the functional effect of a mutation can be predicted from the effect on protein stability. We find that simple sequence-based methods succeed in predicting functional effects of nsSNPs. In fact, such methods consistently outperform approaches that predict functional change through the application of binary thresholds to stability change. We also observed that if stability is affected, functional change is easier to predict than when stability is not affected.

**Conclusion:**

Our results confirmed that stability change is somehow related to function change. However, we also show that the knowledge of stability changes in no way suffices to predict functional changes and that many function changing mutations have no effect on stability.

## Background

Genetic variation is evolution's way of making children adapt better to the environment than their parents. Unfortunately for us, the specific changes in our genetic make-up are more often deleterious than beneficial. When contrasting the concerns of individuals with that of the species we find that most mutations are bad (for the individual) but the diversity created by these mutations helps the species survive. Here, we aim at predicting the effect of each mutation on the particular gene-product. Such predictions could help in addressing problems that originate from the negative perspectives for individuals.

Most of the genetic variation is accounted for by SNPs (single nucleotide polymorphisms). Eleven million SNPs (~11 M) are estimated to be in the human genome [1] (dbSNP release 129 contains already ~15 M entries in human, but only ~6.5 are validated [2]). SNPs vary by their location and effect but can be grouped based on their position in the coding or the non-coding regions of DNA. Furthermore, SNPs resulting in a single amino acid substitution in the translated protein sequence (nsSNPs; non-synonymous SNPs) are differentiated from those that, due to the redundancy of the genetic code, are not. Only ~52 K frequent nsSNPs (> 5% in population) are known in human [3]; a total of 67–200 K nsSNPs is expected [4]. nsSNPs are as much a small subset of all SNPs as all coding nucleotides are of all nucleotides. In analogy, however, we expect that the importance of nsSNPs is as disproportional as that of protein-coding regions to all of DNA. It is therefore not surprising that an increasingly large number of diseases and defects reported in the human mutation databases HGMD [5] and OMIM [6] pertain to nsSNPs. A vast number of all single amino acid substitutions originate from nsSNPs [3]. For simplicity we use the term "nsSNPs" interchangeably with "single amino acid substitutions" in the context of this study.

Not all single amino acid substitutions are deleterious to molecular protein function. By some estimates only 20–30% of the nsSNPs result in an observable functional change [4]. The ability to differentiate disruptive mutations from neutral ones is necessary for a better understanding of protein function. A given nsSNP may disrupt function in two ways: (1) by directly changing the "active" residue (e.g. by replacing the amino acid for a residue involved in ligand binding, catalysis, allosteric regulation, or post-translational modification), or (2) by affecting the scaffolding of the protein (e.g. by deforming and/or destabilizing the binding site or the entire protein structure). Wang and Moult have suggested that disease-associated mutations in the human most often belong to the second class of functional disruptions (in their data set 83% of disease associated mutations affect protein stability) [7].

Structural changes due to mutations can be evaluated in two contexts: (1) as measurable alterations of protein three-dimensional (3D) structure, predicted from crystallography studies or (2) as the changes in protein stability (for instance, measured by unfolding energy; ΔΔG; Eqn. 1).

(1)

Understanding of functional changes due to structural alterations could potentially be derived from either of these contexts. Several methods infer functional effects of nsSNPs based features that include changes in the 3D structure [8-12] and, often, these are more reliable than purely sequence-based approaches [13,14].

Unfortunately, experimental 3D structures are only available for one percent of all proteins. The estimate of reduction in protein stability in terms of unfolding energy changes (Eqn. 1) is experimentally simpler and less expensive to obtain then structure identification, translates directly to a reduced number of folded molecules under normal physiological conditions, and in cases of large changes can be regularly expected to diminish function [15-17]. However, no well-defined algorithm currently succeeds in translating energy changes into functional effects. Such a goal is further complicated by the fact that most single amino acid substitutions result in significant changes to protein stability [18] (this includes at least 30% of the mutations that are not associated with disease [7]). Most often a large destabilization changes or even eliminates function. However, the definition of the word "large" is unclear. In fact, that precise threshold likely differs from one protein to another.

At least one study [19] has attempted to systematically infer functional effects from both known and predicted structural changes. The authors utilized a number of advanced tools and databases to map SNPs to their structural effects in an effort to infer functional alterations. Their method was somewhat successful in identifying functionally important substitutions (albeit less so than the algorithms designed specifically for evaluating functional effects). However, these important results did by no means provide a succinct description of the relationship between changes in protein stability to those in function.

Given that it remains unclear how structural disruptions translate into functional change, we set out to formally evaluate the predictive ability of mutation-associated ΔΔG's on a set of experimentally annotated (both structurally and functionally) single amino acid substitutions. Considering that we were only able to identify a small number of such mutants, we also extracted computational predictions of ΔΔG values for a set of experimentally functionally annotated nsSNPs. We then compared the function-prediction power of ΔΔG to that of directly evaluating changes using sequence-based methods developed specifically for this purpose (SNAP; Screening for Non-Acceptable Polymorphisms [14]; SIFT [13]; Sorting Intolerant from Tolerant; Methods). We find, that in a general case SNAP and SIFT are capable of identifying functional disruptions better than an algorithm using a simple threshold-based binary classification of stability changes.

## Results and discussion

### Stability changes are not easily translated into functional changes

There are at least three reasons why there is no single threshold in ΔΔG stability change at which we are certain that function changes. (1) The threshold at which a mutation is destabilizing enough to disrupt function by reducing the number of folded active proteins depends on the unfolding energy of the wild-type molecule, which ranges from 3–15 kcal/mol [15,20]; i.e. for the inherently more stable proteins a larger change is necessary to significantly alter the concentration of active molecules. (2) Without exact knowing the particular mechanism of protein function, protein destabilization or stabilization events are equally likely to alter function. (3) Destabilizations affecting active sites of the protein may not be manifest in a large ΔΔG, but can still affect function. Keeping these issues in mind we set up an experiment to gauge the resolution limits of binary classification of experimental ΔΔG in predicting nsSNP-associated function changes; i.e. we tried to answer the question of whether there is *one *threshold at which most mutations can be considered deleterious. Alternatively, we would find that the distribution of correct and incorrect functional annotations would be similar throughout the spectrum of possible ΔΔG thresholds. Note, that to address the functional differences between stabilizing and destabilizing mutations, we considered these two types of data points separately.

We collected 66 mutations from the Guerois, *et. al *study [21] (annotated with experimentally derived ΔΔG) which also had functional change annotations in PMD [22,23] (*PMD-exp*; Table 1 and Methods). 54 (82%) mutants were experimentally assigned to have functional effects (non-neutral) and the rest (12 mutants; 18%) were deemed functionally identical to wild type (neutral). The ΔΔG range for this set was from -4.3 to 4.96 kcal/mol. Two of the mutants resulted in functionally delinquent proteins stability-wise identical to wild type (ΔΔG = 0). These were the first examples in our study of absence of one-to-one correspondence between function and structure. Of the 19 stabilizing mutants (ΔΔG < 0 kcal/mol; average ΔΔG = -0.73), 16 (84%) were functionally disruptive. Similarly, of the 45 destabilizing mutants (ΔΔG > 0 kcal/mol; average ΔΔG = 1.67) 36 (80%) affected function.

**Table 1:** Data set summary*.

	*Total*	*FD*	*FSt*	*nFD*	*nFSt*	*Cut*	*FS (%F)*	*FnS*	*nFS (%S)*	*nFnS*
**PMD exp**	64	36	16	9	3	-0.5/0.5	36 (69)	16	8 (19)	4
**PMD all**	3981	2841		1096		Man.	1932 (68)	909	744 (28)	396
**PMD pbd**	1657	849	326	322	160	-0.5/2.5	458 (39)	717	130 (22)	352
**PMD foldx**	810	401	205	126	78	-0.5/1.5	415 (69)	191	109 (21)	95
**PMD 07**	91	51		40		Man.	30 (59)	21	28 (48)	12

* The table summarizes counts of mutants in each data set broken up by effects on function and stability. In column names: (**n**)**F **– (not) functionally disruptive, (**%F**) indicates the percentage of all functionally disruptive mutations in the set, **D **– destabilizing, **St **– stabilizing, (**n**)**S **– (not) structurally disruptive, (**%S**) indicates the percentage of all structurally disruptive mutations. **Cut **is the cutoff in kcal/mol used to determine structural disruption (stabilizing/destabilizing cutoffs;*Man*. means the annotation for the set was manually performed and not described in numbers).

**Figure 1:**
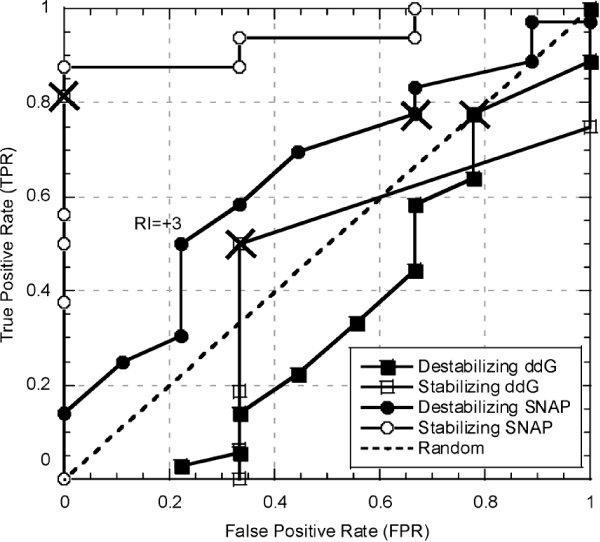
**Function prediction performance of ddG (ΔΔG) and SNAP at various thresholds on *PMD-exp *data set**. We varied the threshold for identifying the mutation as functionally non-neutral from 0.01 to 4 kcal/mol (Methods) separately for both positive (ΔΔG > 0; destabilizing; filled squares) and negative (ΔΔG < 0; stabilizing; open squares) mutants. For SNAP the RI (reliability index) was similarly varied for destabilizing (filled circles) and stabilizing (open circles) mutations from -6 to 6. Peaks (marked by X's) in the ΔΔG curves indicate that reasonable thresholds are 0.5 and -0.5 kcal/mol. Also indicated on the graph are the default SNAP prediction values (RI = 0; marked by X's). Note, that for higher RI (e.g. RI = 3), destabilizing mutations are predicted more accurately.

Given an equal distribution of functionally non-neutral mutations in the stabilizing and destabilizing sets it was clear that the direction of stability change could not alone determine the functional effect. However, the magnitude of the change (the absolute value of ΔΔG) required to alter function could still differ depending on the direction. We utilized various thresholds for the value of ΔΔG to generate a ROC curve mapping structural stability to functional effects (TPR vs. FPR; Eqn. 2; Fig. 1) for both stabilizing and destabilizing samples.

(2)

Our results demonstrate that in general magnitudes of both destabilizing and stabilizing changes are not very informative. For *destabilizing *mutations, using ΔΔG is worse than random (filled squares). At best cutoff of ΔΔG = +0.5 kcal/mol (peak point in filled squares in Fig. 1) 78% of the functionally disruptive destabilizingmutants are identified at 89% accuracy, but only 22% of the functionally neutral mutations are found (at 20% accuracy; Eqn. 2, PA = 89%, PC = 78%, NA = 20%, NC = 22%, Q2 = 67%). Assuming an uninformed guess at the distribution of neutral mutations of 50/50 and the "real data" 80/20 distribution of non-neutrals to neutrals in our set, assures a gain of 9% in accuracy and 28% in coverage of non-neutrals and a 28% loss in coverage of neutrals over random. (Note, if we use the suspected [4] natural distribution of 20–30% non-neutrals for random classification, the same ΔΔG cutoff will generate results with same accuracy, but the gain in non-neutral coverage will come at the cost of more loss in neutral coverage.) For *stabilizing *mutations (open squares, Fig. 1) using a ΔΔG is slightly better than random at peak (cutoff = -0.5 kcal/mol; PA = 89%, PC = 50%, NA = 20%, NC = 67%, Q2 = 53%), but also below random over all. Thus, over the entire set of mutants, using the ΔΔG cutoff of +/- 0.5 kcal/mol results in proper identification of functional change for only (Q2 =) 62% of the data set. These results demonstrate that a binary functional classification of mutations using a ΔΔG cutoff is not very accurate.

### Larger data sets necessary to confirm structure/function correlations

Given the small number of mutations in the *PMD-exp *data set, it is possible that the suboptimal performance demonstrated by ΔΔG in the functional classification is an artifact of the number explosion. To check the validity of our suspicion we needed to collect a larger data set of mutations with known functional and structural effects (ideally reported as ΔΔG). For this purpose, we first extracted 3981 mutants (in 705 proteins) from the PMD that had both annotated stability and functional changes (*PMD-all*; Methods). According to the binary annotation of these mutants, ~71% affected function and ~67% affected protein stability. This distribution of functionally and structurally important mutations was slightly different from that of the *PMD-exp *data set, where 82% affected function, and, at 0.5 kcal/mol cutoff, 54% affected stability. Thus, if the *PMD-exp *classification performance was a statistical error we could expect to see improvement for this data set.

Both the structural and the functional changes recorded in PMD are in qualitative format (instead of ΔΔG; Methods). Using this form of annotation we could only generate one measurement of usefulness of stability changes in predicting functional ones (stability change = function change; PA = 73%, PC = 68%, NA = 30%, NC = 36%, Q2 = 59%). This performance was even worse than was expected from *PMD-exp *results, but the comparison was not exact; i.e. we could not search for an optimal cutoff in a binary classification of stability change. To use *PMD-all *more directly for comparison with the *PMD-exp *we used FoldX [21], a structure based program for energy calculations in proteins, to annotate the mutants in the *PMD-all *set with ΔΔG values. FoldX predictions of ΔΔG changes due to single amino acid substitutions were found in a previous study to be very well correlated with the experimental energy changes [21]. However, since FoldX requires the presence of a known wild-type protein 3D-structure we were limited to a subset of only those substitutions for which this structure was available (*PMD-pdb*; Methods). This set contained 1657 mutants in 232 PDB structures (~75% functionally and ~71% structurally important; distribution very similar to *PMD-all*).

### Some energy predictions agree with expert annotations of stability

**Figure 2:**
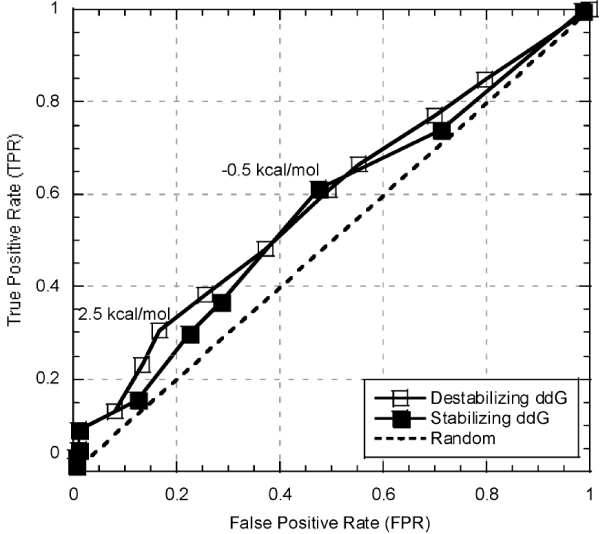
**Correlation of PMD annotation and FoldX predictions**. To gauge the best ΔΔG cutoff for annotating a mutation as neutral stability-wise we correlated PMD binary annotations with FoldX predictions. Overall correlation was relatively weak, pointing out the subjectivity of annotations, possible problems of curation, and FoldX prediction errors. The best correlation between binary annotation and FoldX predictions was at -0.5 kcal/mol for stabilizing mutations (open squares) and 2.5 kcal/mol for destabilizing ones (filled squares).

PMD is a literature-based database that relies on human assessment of data reported in literature for identifying effects of mutations. Both the report and the interpretation of data are subjective opinions of the corresponding people. To assure the cleanliness and quality of our data, we correlated the binary annotations of stability changes reported in PMD with the FoldX predictions for these proteins (Fig. 2). Because of the nature of the binary annotations in PMD we did not expect a perfect correlation between the predictions and these classifications at any threshold of ΔΔG. As Fig. 2 illustrates, the correlation between binary annotations and actual magnitudes of ΔΔG was relatively weak. Classification was most exact (furthest from random) for destabilizing mutations at ΔΔG = 2.5 kcal/mol and for stabilizing mutations at ΔΔG = -0.5 kcal/mol. At this cutoff there were 810 (~49% of 1657) correct annotations. Note, given the curve shape, other cutoff points could be reasonably chosen, but subsequent measurements reported here were similar for other tried values.

Given the weakness of the correlation between the experimental data reported in PMD and the predictions from FoldX, we needed to identify a subset of our data that could be trusted in for attributing correct functional and structural changes to specific mutations. For this purpose, we retained for further testing only those mutations that were correctly classified by PMD reports at our chosen FoldX cutoffs (*PMD-foldx; *Methods). This selection assured that subjective study opinions (or errors in manual annotation) reflected in PMD entries corresponded to signals that could potentially be picked up using a predicted (possibly erroneously) ΔΔG threshold; i.e. if reports of stability changes agreed between expert annotation (PMD) and prediction (FoldX), both could be expected to be "biologically" correct. Of the mutants in the resulting data set ~75% altered function and ~57% altered the stability of the protein.

### Larger data set confirms difficulty in converting energy changes to function changes

**Figure 3:**
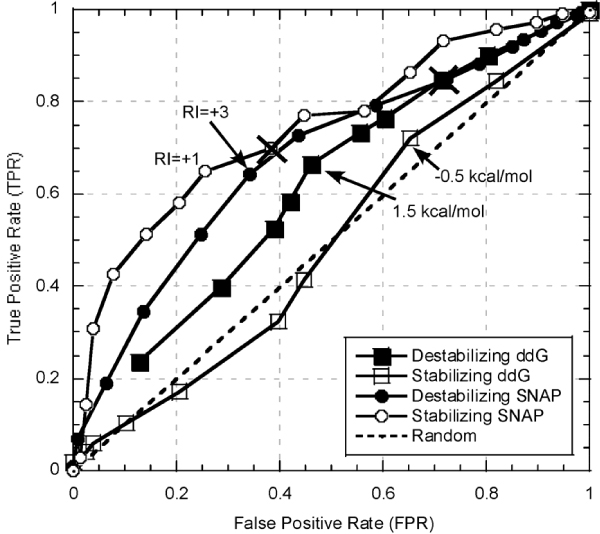
**Function prediction performance of FoldX predicted ddG (ΔΔG) and SNAP at various thresholds on *PMD-foldx *data set**. We varied the threshold for identifying the mutation as functionally non-neutral from 0.01 to 4 kcal/mol (Methods) separately for both positive (ΔΔG > 0; destabilizing; filled squares) and negative (ΔΔG = < 0; stabilizing; open squares) mutants. For SNAP the RI (reliability index) was similarly varied for destabilizing (filled circles) and stabilizing (open circles) mutations from -8 to 8. Peaks in the ΔΔG curves indicate that reasonable thresholds are -0.5 and 1.5 kcal/mol. Also indicated on the graph are the default SNAP prediction values (RI = 0; marked by X's). Note, that for higher RIs (left side of the graph), mutations are predicted more accurately.

Applying ΔΔG thresholds to *PDB-foldx *we mapped the results to a ROC curve (Fig. 3). Like in *PMD-exp*, using ΔΔG to annotate functional changes of stabilizing mutants of the *PMD-foldx *data resulted in performance that was only slightly better than random at peak point. For destabilizing mutants, the chosen thresholds gave better results throughout the curve. Yet in terms of overall accuracy these still translated to low numbers. For instance, at -0.5 kcal/mol cutoff (stabilizing peak, Fig. 3) only 62% of the stabilizing mutants were correctly identified as functionally disruptive (Q2; Eqn. 2; in *PMD-exp *Q2 = 53%). At 1.5 kcal/mol cutoff (destabilizing peak, Fig. 3) the number of correct predictions was 64% (Q2; Eqn. 2; in *PMD-exp *Q2 = 67%). At these cutoffs, overall accuracy for *PMD-foldx *(Q2 = 63%; Eqn. 2) was only slightly higher than that of *PMD-exp *at its optimal cutoff (+/- 0.5 kcal/mol, Q2 = 62%). This poor performance once again confirmed the difficulty in translating stability changes directly into functional ones.

### Computational methods are better at identifying functional effects of mutations

The problems with using energy thresholds for identifying functional changes suggest taking a different route to this type of classification. For instance, using SNAP [14] or SIFT [13] (Methods), methods specifically designed for evaluating functional consequences of single amino acid substitutions from sequence, we were able to obtain as good and better prediction performances. SNAP outperformed using an energy threshold throughout most of the spectrum of accuracy/coverage values for both destabilizing and stabilizing examples of the *PMD-exp *(Fig. 1) and the *PMD-foldx *(Fig. 3) data sets. As expected, accuracy was better at higher RI values (i.e. more reliable predictions), but even using default cutoffs produced better overall results. Similarly, SIFT did better then ΔΔG (but slightly worse then SNAP; at cutoffs described: *PMD-exp *Q2 ΔΔG = 62%, SNAP = 73%, SIFT = 63%; *PMD-foldx *Q2 ΔΔG = 63%, SNAP = 70%, SIFT = 68%; Eqn. 2). Interestingly, of mutations with altered stability, SNAP correctly assigned functional changes to 73%, while only 65% of the ones with unchanged stability were classified properly. This result suggests that mutations with significant structural alterations are easier to differentiate in terms of their effect on function then the ones that remain structurally intact.

### Accounting for overlap with training data confirms accuracy of performance estimates

**Figure 4:**
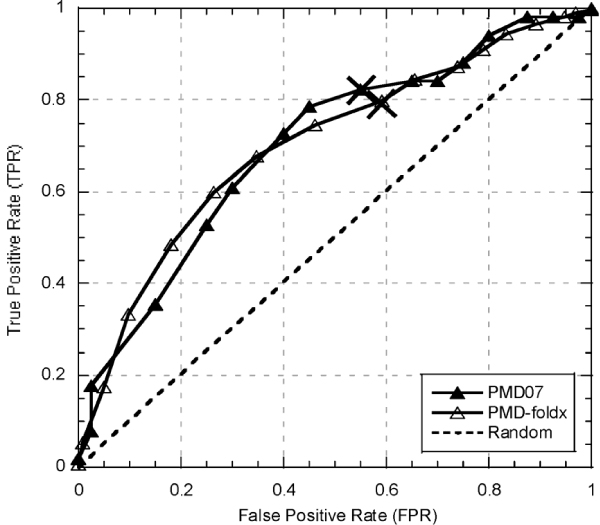
**SNAP performance for data not seen in training (*PMD07*)**. SNAP predicted the effects of mutations that it has not encountered in training (*PMD07*, filled triangles) just as accurately (at default cutoff RI = 0; marked by X's) as the mutations in the larger *PMD-foldx *set (open triangles). This result suggests that effects of *PMD-foldx *mutants were not memorized in the course of SNAP training.

In evaluating the performance of SNAP on the PMD-based data sets, it is important to note that the method is neural-network based (Methods) and was originally trained on the PMD data. In testing artificial learning devices, it is proper procedure to separate training and testing data points to avoid over-estimates of performance through memorization of samples. Such a split was not possible in our case since the data sets inherently overlapped. While, SNAP has been shown in prior testing to demonstrate a better performance than the one reported here (original Q2 reported ~79% [14]) it was still proper to exercise caution with these results. To make sure that we did not overestimate SNAP's prediction accuracy we extracted from the most recent version of PMD available (*PMD07*, Methods) the mutants that were not present in the training PMD data set (91 mutants in 38 proteins; 56% functionally non-neutral). We then measured SNAP's performance on this data to find that mutations in this set were annotated just as accurately as the ones in *PMD-foldx *(Fig. 4). These results confirm the validity of performance numbers reported for all the PMD-based data sets in this study.

### Protein stability and function are correlated but not equivalent

Overall, our results are in line with earlier findings from Wang *et al *[7], Steward *et al *[24] and DePristo *et al *[18] which suggest that while many functionally disruptive mutations are due to structural changes, a fairly large segment of functionally neutral mutants is also structurally disruptive. For instance, in the *PMD-exp *set 69% of mutations affecting function damaged the structure while 19% of the structurally disruptive mutants did not affect function. Trends of function/structure disruption correlation were similar for all data sets except *PMD-pdb *where 61% of the functionally deleterious mutations were not associated with a structural change (Table 1). This difference, however, can be attributed to a high threshold of considering a destabilizing substitution to be structurally disruptive.

From the data presented above it is clear that functional changes due to mutations do not always correspond to changes in stability. This concept is not novel, nor is it particularly surprising biologically. For instance, mutations eliminating binding residues do not have to be very destabilizing to be damaging while promiscuity of enzyme binding sites that associates with destabilization may not reflect on function. In our data sets we found quite a few examples of both stability-neutral/function-non-neutral mutants and vice versa. For example, mutagenesis studies [25] of the carboxyl tail of the mouse PKA (protein kinase A; SwissProt [26,27] id: KAPCA_MOUSE) have shown that a tyrosine in position 330 of the protein is very important for maintaining kinase activity but not its structural integrity. Thus, many substitutions at this position yield structurally normal, yet functionally delinquent molecules. On the other end of the spectrum, there is the alanine to leucine mutation in position 172 of the 3-isopropylmalate dehydrogenase in *T. thermophilus *(SwissProt id: LEU3_THETH). This mutation affects the interdomain interface of the enzyme and produces a much more closed conformation of the molecule (which, incidentally, is a lot more stable). However, the substitution does not affect the domains' ability to move as necessary to maintain wild-type activity [28].

These examples are just some of the many that fall into a category of mutations that would always be misclassified by a "stability only"-based algorithm. Interestingly, a major novel finding of this study is that only about a third of mutations fall into this category; i.e. about two thirds of all mutants can in fact be correctly classified for their functional effects by considering the associated stability changes. On the other hand, we also find that functional classification is more precise using a computational method specifically developed for this purpose. The latter is also more advantageous for proteins with minimal information available (i.e. only sequence). Another surprising finding is that functional annotation of mutations that are disruptive stability-wise is simpler then when no stability changes are involved. Overall, we believe that the knowledge of protein stability/function correlation gleaned from this work will contribute significantly to the understanding of the field and to the development of algorithms capable of identifying functional importance of nsSNPs.

## Conclusion

We collected experimental and computationally derived information regarding the effects of single amino acid substitutions on protein stability and function. For each of the available data sets we predicted functional effects of mutations using SNAP and SIFT, computational methods developed specifically for this purpose, and using stability alterations reported as changes in unfolding energy of the protein (ΔΔG). Comparing the predictive abilities of both approaches we find that, for our data, SNAP and SIFT perform better than using a binary threshold of ΔΔG. These results suggest that there is no simple relation that associates protein stability change to protein function change. In fact, for about a third of the mutants, these two features appear uncorrelated. Mutations that affect stability are better differentiated in terms of their effect on function than those that do not affect stability. Future implementations of computational algorithms could therefore benefit from using stability information, where available, in making predictions of functional effects of mutations.

## Methods

### PMD data

PMD (Protein Mutant Database) [22,23] is a literature-based database containing experimentally derived annotations of changes in protein function and/or structure due to mutation. For the purposes of this study we extracted from PMD only those entries describing single amino acid substitutions. Changes in function and structure are reported in PMD in a qualitative form ([---] means significant decrease in function/stability, [=] means no change, and [+] stands for increase in functionality/stability, etc.). Of the set extracted above we chose only those entries containing an annotated functional change (FUNCTION field) and/or annotated stability change (STABILITY field). Changes in function and stability were recorded in a binary format (neutral = identical to wild type; non-neutral = different from wild-type). If two studies of the same mutant differed in their annotations, the effect of the substitution was recorded as non-neutral. The version of PMD used to train SNAP and to extract the PMD-exp,-all,-pdb,-foldx data sets was created in Dec. 2005.

### PMD-exp mutants

We took from the Guerois *et. al*. study [21] all single amino acid substitutions that have been experimentally annotated with ΔΔG (Eqn. 1). We used BLAST [29] for each of the proteins in this set to obtain alignments (at 100% sequence identity) to the sequences annotated with functional changes in the PMD database. We recorded functional effects of mutations from PMD corresponding to mutations in the Guerois et al data set for all aligned sequences to make the *PMD-exp *data set.

### PMD-all mutants

We extracted from PMD a set of all entries containing experimental annotations of their effects on structural stability of the affected protein (STABILITY field) *and *on its function (FUNCTION field) to make the *PMD-all *data set

### PMD-pdb mutants

FoldX [21] is a computational method that uses experimental protein structure data to estimate the value of all atomic interactions on the stability of said protein. Using these values FoldX can calculate the effect of mutation on protein stability. To obtain FoldX predictions of mutant ΔΔG's we extracted from the *PMD-all *data set all mutants in sequences mapping to a PDB id to generate the *PMD-pdb *data set. The mapping was accomplished by intersecting PMD PDB annotations of the selected entries with BLAST alignments of PDB chains to the reported PMD sequences (at ≥ 97% sequence identity). If the mutated residue reported in the PMD entry differed from the PDB chain residue at the same position, the entry was discarded from the data set.

### PMD-foldx mutants

We selected from the *PMD-pdb *data set only those mutations that agreed (at ΔΔG = -0.5/2.5 kcal/mol cutoffs) with the binary annotations in PMD; i.e. we selected only those mutants that were annotated as neutral in PMD and had a FoldX prediction of > -0.5 and < 2.5 kcal/mol or those that were non-neutral according to PMD and had a FoldX prediction < = -0.5 or > = 2.5 kcal/mol.

### PMD07 mutants

We extracted mutations from the new version of PMD (created in Mar. 2007) in the same manner as was applied to create *PMD-foldx *(we used the same cutoffs of -0.5 and 2.5 kcal/mol to identify mutations for which expert annotations agreed with FoldX predictions). To test accuracy of SNAP on novel samples we collected from this set only those mutations that were not present in the original *PMD-foldx *set.

### Stabilizing and destabilizing mutations

In *PMD-exp *data set two mutations, for which ΔΔG = 0, were not considered as part of either stabilizing or destabilizing data set. In all other sets, we classified as stabilizing those mutations where predicted ΔΔG < = 0 and as destabilizing those where ΔΔG > 0.

### Structure-function correlation

To evaluate the correlation of the structural and functional effects we varied the threshold of ΔΔG at which a mutation is assigned to be functionally neutral. We had considered cutoffs of 0.01, 0.3, 0.5, 0.8, 1.0, 1.5, 2.0, 2.5, 3.0, 3.5, and 4.0 kcal/mol in both stabilizing and destabilizing directions.

### SNAP predictions

SNAP [14] is a neural network based method for identifying from sequence functionally disruptive single amino acid substitutions. The inputs to SNAP include secondary structure and solvent accessibility predictions, evolutionary and family information, biophysical differences between the wild type and mutant amino acids, statistical likelihoods of observing residue triplets around the mutation site, SIFT [13] predictions, and SwissProt [26] annotations (if available). SNAP outputs both a binary prediction (neutral/non-neutral) and a reliability index (RI; 0–9) representative of the likely accuracy of prediction. For all mutants in the *PMD-exp*, *PMD-foldx*, and *PMD07 *data sets we ran SNAP and recorded both the binary predictions and the RI. SNAP is generally more accurate at higher RI. To obtain the ROC curves, we dialed through the RI values (in both positive/non-neutral and negative/neutral directions) as the threshold for assigning a neutral prediction.

### SIFT predictions

SIFT [13] is a sequence based method that uses sequence homology and biophysical amino acid similarity to predict functional effects of single amino acid substitutions. SIFT outputs both a binary prediction (tolerated/deleterious) and a score (0–1, where score < = 0.05 means that the mutation is deleterious). SIFT scores are not meant to be used as prediction accuracy estimators. For all mutants in *PMD-exp *and *PMD-foldx *data sets we ran SIFT and recorded the binary prediction (tolerated/deleterious). For some of the mutants (4 in PMD-exp and 25 in PMD-foldx) we were unable to obtain SIFT predictions. For these instances a random prediction was generated (50/50 chance of being classified as tolerated or deleterious).

## Abbreviations used

SNAP: Screening for Non-Acceptable Polymorphisms; SIFT: Sorting Tolerant From Intolerant; PMD: Protein Mutant Database; PDB: Protein Data Bank; nsSNP: non-synonymous Single Nucleotide Polymorphism;

## Competing interests

The authors declare that they have no competing interests.

## Authors' contributions

YB conceived and carried out the study and drafted the manuscript. BR participated in its design and helped to draft the manuscript. All authors read and approved the final manuscript.
